# Featured Gut Microbiomes Associated With the Progression of Chronic Hepatitis B Disease

**DOI:** 10.3389/fmicb.2020.00383

**Published:** 2020-03-20

**Authors:** Zhangran Chen, Yurou Xie, Fei Zhou, Bangzhou Zhang, Jingtong Wu, Luxi Yang, Shuangbin Xu, Robert Stedtfeld, Qiongyun Chen, Jingjing Liu, Xiang Zhang, Hongzhi Xu, Jianlin Ren

**Affiliations:** ^1^Department of Gastroenterology, Zhongshan Hospital Xiamen University, Xiamen, China; ^2^Institute for Microbial Ecology, School of Medicine, Xiamen University, Xiamen, China; ^3^College of Chemistry and Chemical Engineering, Xiamen University, Xiamen, China; ^4^School of Life Sciences, Xiamen University, Xiamen, China; ^5^Fisheries College, Jimei University, Xiamen, China; ^6^Civil and Environmental Engineering, Michigan State University, East Lansing, MI, United States

**Keywords:** hepatitis B virus, gut dysbiosis, cooccurrence network, liver cirrhosis, random forest

## Abstract

Dysbiosis of gut microbiota during the progression of HBV-related liver disease is not well understood, as there are very few reports that discuss the featured bacterial taxa in different stages. The aim of this study was to reveal the featured bacterial species whose abundances are directly associated with HBV disease progression, that is, progression from healthy subjects to, chronic HBV infection, chronic hepatitis B to liver cirrhosis. Approximately 400 fecal samples were collected, and 97 samples were subjected to 16S rRNA gene sequencing after age and BMI matching. Compared with the healthy individuals, significant gut microbiota alterations were associated with the progression of liver disease. LEfSe results showed that the HBV infected patients had higher *Fusobacteria*, *Veillonella*, and *Haemophilus* abundance while the healthy individuals had higher levels of *Prevotella* and *Phascolarctobacterium.* Indicator analysis revealed that 57 OTUs changed as the disease progressed, and their combination produced an AUC value of 90% (95% CI: 86–94%) between the LC and non-LC groups. In addition, the abundances of OTU51 (*Dialister succinatiphilus*) and OTU50 (*Alistipes onderdonkii*) decreased as the disease progressed, and these results were further verified by qPCR. The LC patients had the higher bacterial network complexity, which was accompanied with a lower abundance of potential beneficial bacterial taxa, such as *Dialister* and *Alistipes*, while they had a higher abundance of pathogenic species within Actinobacteria. The compositional and network changes in the gut microbiota in varied CHB stages, suggest the potential contributions of gut microbiota in CHB disease progression.

## Introduction

Hepatitis B virus (HBV) infection is a global epidemic public health issue (3.5% of the global population have been infected), which may be accompanied by ongoing low-grade liver inflammation (Lavanchy et al., 2004; Ott et al., 2012). HBV infection progresses through several clinical phases depending on liver injury: chronic HBV infection (HBVI), chronic hepatitis B (CHB) and liver cirrhosis (LC).

The gut microbiota is the largest group of microorganisms living in the human body and is composed of bacteria, archaea, virus fungi, and protist; the microbiota has been found to be closely related to health (Ray, 2012; Tripathi et al., 2018). Because of the gut-liver circulation *via* the gut-microbiota-liver axis, nutrients, bacterial products/toxins and metabolites from the gut enters the liver, which play important roles in the progression of liver disease (Wiest et al., 2017). Previous studies indicate that compositional changes of the gut microbiota are closely related to chronic liver diseases, such as non-alcoholic fatty liver disease (NAFLD) (Bibbò et al., 2018; Hoyles et al., 2018; Ponziani et al., 2018), non-alcoholic steatohepatitis (NASH) (Yamada et al., 2017; Bomhof et al., 2018; Ye et al., 2018), alcoholic liver disease (ALD) (Hartmann et al., 2018; Shao et al., 2018; Stärkel et al., 2018), hepatic encephalopathy (HE) (Tilg et al., 2016; Mancini et al., 2018), cirrhosis (Garcia-Tsao and Wiest, 2004; Bajaj et al., 2018a; Guo et al., 2018), hepatocellular carcinoma (HCC) (Zamparelli et al., 2017), and HBV infection. Increasing evidence suggests that the gut microbiota has evolved as a new important player in the pathogenesis of hepatitis B virus-induced chronic liver disease. A variety of gut commensals, such as *Faecalibacterium prausnitzii*, *Enterococcus faecalis*, Enterobacteriaceae, *Bifidobacteria*, and lactic acid bacteria (*Lactobacillus*, *Pediococcus*, *Leuconostoc*, and *Weissella*), are known to be different in the intestine of HBV cirrhotic patients (Lu et al., 2011). We have previously induced hepatitis B virus e-Antigen (HBeAg) clearance in patients with positive HBeAg after undergoing long-term antiviral therapy through several fecal microbiota transplantation (FMT) treatments, which were accompanied by significant changes in the microbiota composition (Ren et al., 2017). However, how microbiome profiles change during the transition from healthy to HBVI, CHB, and LC conditions remains to be further investigated to determine potential therapeutic microbiome-targets in the future that can reverse or remiss HBV infections to less severe conditions. Consequently, the systematic monitoring of HBV infected patients to reveal unique bacterial taxa that can postpone disease course during severe hepatic deterioration, is of great importance.

Based on the above consideration, our study focused on the gut microbiota of 21 healthy individuals and 76 chronic HBV infected patients with HBVI, CHB, or LC through high throughput 16S rRNA gene sequencing. Our objectives were to: (i) elucidate shifts in the microbial community structure and diversity along with the HBV infection process, (ii) determine indicator and differential bacterial taxa for HBV infection progression and the drivers that correlate with them and test the existence of them in clinical samples, and (iii) probe into the gut microbial network features that are associated with disease stages.

## Materials and Methods

### Clinical Trial Number

This project was approved by the Ethics Committee of Xiamen University (Permit Number ID: XMU-IRB-2018001) and registered in ClinicalTrials.gov (ID: NCT03587467). This study was performed in accordance with the approved guidelines. All participants provided informed consent, and the study protocol conformed to the ethical guidelines of the Declaration of Helsinki (World Medical Association, 2008).

### Healthy Individuals

A total of 21 age- and BMI-matched Chinese healthy individuals were enrolled at Zhongshan Hospital, affiliated with Xiamen University (Xiamen, China), from December 2017 to May 2018. The inclusion criteria were as follows: (a) alcohol free history or alcohol consumption less than 140 g per week in males, less than 70 g per week in females; (b) smooth and soft stool that was sausage or snake shaped, and (c) voluntary participation in this study. The exclusion criteria were as follows: (a) symptoms of digestive system disorders, such as hematochezia, constipation, abdominal distention, abdominal pain, diarrhea, and jaundice within 1 month; (b) abnormal results of several tests, including: routine blood, liver function, renal function, blood fat, fasting blood glucose, HBsAg, routine fecal and fecal occult blood tests; (c) an enteritis diagnosis within 1 month; (d) chronic obstructive pulmonary disease, renal insufficiency and other systemic diseases; (e) autoimmune disease; (f) chronic fatigue syndrome and neuropsychic disease; (g) a history of antibiotic, microecological preparation, gastrointestinal motility medicine, laxative, weight loss drug, glucose lowering, blood fat regulation, glucocorticoid, or immunosuppressant treatment within 1 month; (h) history of organic diseases in the digestive system, such as gastrointestinal polyposis, ulcers, cirrhosis, and malignancies; (i) history of gastrointestinal surgery; or (j) a family history of diabetes, hypertension, coronary heart disease, metabolic syndrome, etc.

### Enrolled Patients and Study Design

A total of 76 age and BMI-matched patients with chronic hepatitis B virus (HBV) infection were enrolled at Xiamen University (Xiamen, China) from December 2017 to May 2018. As HBV infection progresses chronically through several clinical stages that are determined by liver damage, the HBV infected patients were grouped into HBV infection (HBVI, *n* = 23), chronic hepatitis B (CHB, *n* = 28) and liver cirrhosis (LC, *n* = 25) groups. The inclusion criteria for these groups were (a) alcohol free history or alcohol consumption less than 140 g per week in males and, less than 70 g per week in females; and (b) they meet the diagnostic criteria for chronic hepatitis B according to the “EASL 2017 Clinical Practice Guidelines on the management of hepatitis B virus infection.” The exclusion criteria for the HBVI, CHB and LC groups were: (a) a history of antibiotic, microecological preparation, gastrointestinal motility medicine, weight loss drug, glucose lowering, blood fat regulation, and glucocorticoid or immunosuppressant treatment within 1 month; (b) other causes of liver disease such as NAFLD, autoimmune liver disease, hepatitis A, hepatitis C, hepatitis D, hepatitis E and liver parasite infection; (c) chronic fatigue syndrome and neuropsychic disease; (d) a history of gastrointestinal surgery; or (e) a family history of diabetes, hypertension, coronary heart disease, metabolic syndrome, etc. The LC patients received the following tests: computed tomography (CT) alone (*n* = 9), magnetic resonance imaging (MRI) alone (*n* = 2), ultrasonography alone (*n* = 3), CT plus MRI (*n* = 3), CT plus ultrasonography (*n* = 3), MRI plus ultrasonography (*n* = 1), CT plus MRI plus ultrasonography (*n* = 4).

### Fecal Sample Collection and DNA Extraction

Fecal samples from healthy individuals and HBV infected patients were collected on the day of the medical examination and immediately frozen at −80°C. DNA was extracted from approximately 0.25 g of the fecal samples using the QIAamp PowerFecal DNA Kit (Qiagen, DE) according to the manufacturer’s instructions^1^. The purity and concentration of the isolated DNAs were assessed using spectrophotometry (Multiskan^TM^ GO, Thermo Fisher Scientific, United States). The DNA extracts were also evaluated for quality using agarose (1.5%) gel electrophoresis in 1 × Tris-Acetate-EDTA buffer. DNA samples were stored at −20°C before being used as templates for next-generation sequencing library preparation.

### PCR Amplification and Sequencing Library Preparation

The V3-V4 hypervariable region of the bacterial 16S ribosomal RNA (rRNA) gene was amplified from the DNA samples with the barcoded forward primers 341F (5′-CCTACGGGNBGCASCAG-3′) and the reverse primers 806R (5′-GGACTACNVGGGTWTCTAAT-3′) using KAPA HIFI HotStart ReadyMix (KAPA Biosystems, United States). The PCR thermocycler conditions were as follows: initial denaturation at 95°C for 3 min; 30 cycles of denaturation at 95°C for 20 s, annealing at 60°C for 30 s, and extension at 72°C for 30 s; and a final extension at 72°C for 10 min. The PCR products were purified using an AxyPrep PCR Cleanup Kit (Axygen, United States) according to the manufacturer’s protocol. The purified PCR products were eluted, and their DNA concentrations were determined with a Qubit 3.0 Fluorometer (Thermo Fisher Scientific Inc., Waltham, MA, United States) in conjunction with the Qubit dsDNA HS Assay Kit (Invitrogen, CA, United States). Equal amounts of each amplified sample were then enriched in a normalization step and pooled. They were pooled in equimolar amounts and then paired-end sequencing was performed by the Beijing Novogene company^2^ on a HiSeq 2500 (Illumina, San Diego, CA, United States) in rapid mode with v2 reagents which produced 250 bp reads per end, according to manufacturer’s instruction.

### Data Processing of the 16S rRNA Gene Sequences

The raw paired-end reads were assembled and merged by FLASH (Mago and Salzberg, 2011) under default parameters with the –x 0.2 and –M 200 for V3-V4 region, and then the PCR primers were subsequently truncated by cutadapt (Martin, 2011). Those sequences with low quality and multiple N bases were eliminated with a setting parameter of an error rate less than 0.1. The quality-controlled sequences were further chimerically removed and OTU clustered by Usearch (Edgar, 2013). In detail, all reads were demultiplexed into one file, clustered at 97% similarity, then the chimera checking was performed using UCHIME in reference mode. Representative sequences were generated and singletons were removed, and a final OTU table was created. The representative sequences of OTU were aligned on the Greengenes database for taxonomic classification by RDP Classifier. All bioinformatics analyses were performed at the national supercomputer center in Guangzhou, China.

### Quantitative Real-Time PCR of *Dialister succinatiphilus* and *Alistipes onderdonkii*

The full length of the 16S rRNA genes of OTU51 (*D. succinatiphilus*) and OTU50 (*A. onderdonkii*) (including all strains belonging to the two species) were selected to design and test the primers with Primer-BLAST^3^. Primers that only targeted the objective bacteria were chosen and later verified by Sanger sequencing of the PCR products that were amplified with the primers; then, these primers were used for further real-time qPCR analysis. In brief, DNA from 32 healthy and HBV infected patients was extracted and adjusted to a concentration of 20 ng/μl. The real-time PCR was carried out using a TB^TM^ Green Premix Ex Taq^TM^ II Kit (TaKaRa Biochemicals, China) in 10 μl volumes, which included 5 μl TB Green Premix Ex Taq II (2×), 0.4 μl forward and reverse primer and 0.8 μl DNA. The 16S rRNA gene was used to standardize the results by eliminating variation in the quantity and quality. All reactions were performed with three technical replicates on a CFX384TM Real-Time System (Bio-Rad). The qPCR program consisted of denaturation at 95°C for 30 s and 40 cycles at 95°C for 5 s and at 60°C for 30 s. The relative gene expression abundance was quantified using the 2^–ΔΔCt^ method.

### Statistical Analyses

Alpha-diversity metrics, including Shannon, Simpson, Chao1, and Pielou’s evenness (J), and β-diversity metrics, including Bray–Curis distance with vegdist, PERMANOVA with the adonis function, and the envfit function, were all performed with the R package vegan (version 2.5.3) (Oksanen et al., 2015). The shared OTUs were calculated and visualizing using the R package VennDiagram (version 1.6.20) (Boutros and Chen, 2011). The taxa abundance was measured and plotted using ggplot2 (version 3.1.0) (Wickham, 2009). LEfSe analysis was performed to identify taxa with differentiating abundance in the different group with LEfSe1.0 (Segata et al., 2011). ANOVA tests were either conducted by a oneway.test and pairwise.t.test with the *P*-value adjusted to the “BH” method for multiple comparisons or the bartlett.test using the equal variance and LDuncan method (package laercio, 1.0-0) to group differences. Moreover, the indicator analysis (singleton and doubleton genera removed first) was conducted using the R package indicspecies (version 1.7.1) (De Caceres and Legendre, 2009) with 999 permutations, and the *P*-values were corrected for multiple comparison using the R package qvalue (Dabney and Storey, 2015) with a false discovery rate of 10%. Finally, the results were visualized using a custom R script based on ggplot2. Significant correlations between the abundances of indicator species and clinical properties were calculated by evaluating Pearson’s correlations with the cor and cor.test function in the R package stats. Cooccurrence network pattern analyses were made by Gephi 0.9.1 (Cherven, 2015). These analyses were performed using R3.5.1 (R Core Team, 2014). We performed random forest classification using the implementation of this method in Python’s scikit-learn package^4^. We used a specified group as the positive group, while the others were negative. For example, first, the LC group was regarded as the positive group, the others were regarded as the negative group. Then, we conducted a 10-fold cross-validation analysis and estimated the classifier accuracy of the random forest model. The distribution of indicator species with the disease progression were made by Graphapad Prism 5.

### Data and Code Availability

The 16S sequencing raw reads of this HBV study are available on the NCBI SRA. The accession number for the 16S sequencing data reported in this paper is NCBI SRA: PRJNA558158. The brief pipeline for the 16S rRNA gene sequence analysis and the main data sets used during the current study are available on GitHub^5^.

## Results

### Gut Microbial Community Shift Across HBV Infection Status

**FIGURE 1:**
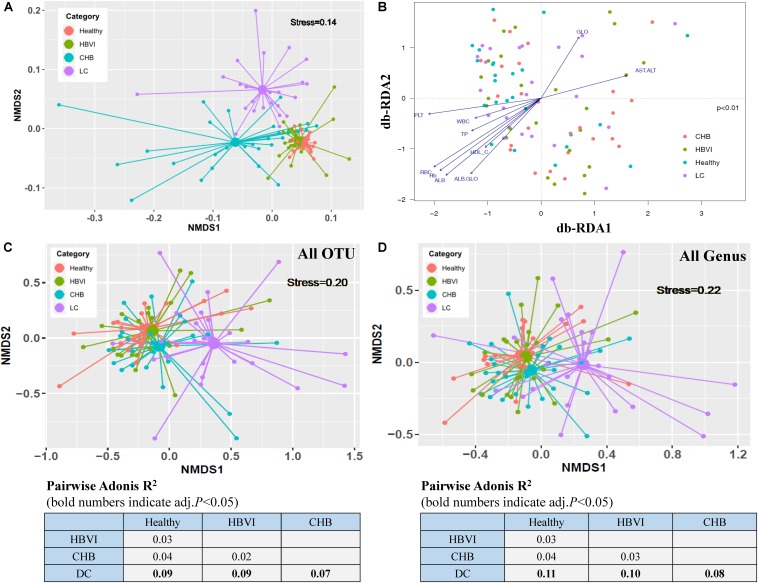
Ordination analyses of clinical parameters and gut bacterial community grouped by disease progression. **(A)** Non-metric multidimensional scaling (NMDS) based on the Bray–Curtis distance showing the overall distribution pattern of clinical parameters. **(B)** db-RDA analyses reflecting differences in gut microbiota structures fitted with significantly correlated clinical properties. The letter with arrows indicated the total variance explanation degree. **(C)** Non-metric multidimensional scaling (NMDS) plot based on Bray–Curtis distances calculated using OTU compositions. **(D)** NMDS plot based on Bray–Curtis distances calculated using genus compositions. Adonis *R*^2^ (effect size) between groups of different category subjects was calculated with 999 permutations and shown in the lower part of the figure. HBVI, chronic HBV infection; CHB, chronic hepatitis B; and LC, liver cirrhosis. OTU, operational taxonomic unit. The color scheme for Healthy, HBVI, CHB and LC in the graph are red, green, blue, and purple.

To determine associations between microbiome profiles and HBV infection status, we performed 16s rRNA gene sequencing on the stool samples collected from healthy subjects (*n* = 21) and HBVI (*n* = 23), CHB (*n* = 28) and LC (*n* = 25) patients (Table 1). The average raw reads (143,149), clean reads (122,586), off-chimera reads (110,422), and OTU numbers per patient were 236 (Supplementary Table S1). The differences between the clinical parameters were displayed *via* NMDS ordination (Figure 1A), which showed differences between clinical properties and communities among the healthy, CHB, and LC subjects. Regarding gender, there were no obvious differences among HBVI, CHB, and LC subjects, compared with the healthy individuals (Supplementary Figure S1A). A significant increase in serum total bilirubin (TBIL), ALT, and AST levels only occurred in the CHB patients (Table 1).

**TABLE 1:** Baseline demographics and clinical characteristics.

	Healthy	HBVI	CHB	LC
Sample size	21	23	28	25
Sex (Male/Female)	16/5	16/7	19/9	20/5
Age	45.14 ± 9.03	43.83 ± 11.76	44.71 ± 11.3	51.24 ± 6.91
BMI (kg/cm^2^)	21.97 ± 2.91^ab^	23.37 ± 3.93^a^	23.21 ± 3.54^ab^	21.87 ± 2.61^b^
WBC count(10^9^/L)	6.06 ± 1.15^a^	5.87 ± 1.48^a^	5.48 ± 1.41^a^	4.08 ± 1.92^b^
RBC count(10^12^/L)	5 ± 0.46^a^	4.55 ± 0.63^b^	4.4 ± 0.68^b^	3.92 ± 0.81^c^
Hb(g/L)	150.05 ± 11.23^a^	137.78 ± 21.62^ab^	134.75 ± 21.96^b^	115.52 ± 25.91^c^
PLT count(10^9^/L)	235.68 ± 41.04^a^	227.04 ± 55.87^ab^	199.5 ± 55.77^b^	84.76 ± 52.78^c^
TP(g/L)	73.28 ± 4.78^a^	68.82 ± 9.82^b^	66.71 ± 5.63^b^	67.36 ± 6.58^b^
ALB(g/L)	48.87 ± 3.49^a^	43.27 ± 7.4^b^	38.51 ± 5.13^c^	35.16 ± 5.24^d^
GLO(g/L)	24.41 ± 3.99^a^	25.55 ± 4.23^ab^	28.2 ± 4.53^b^	32.19 ± 6.06^c^
ALB/GLO	2.06 ± 0.44^a^	1.71 ± 0.31^b^	1.4 ± 0.29^c^	1.14 ± 0.29^d^
TBIL(μmol/L)	7.7 ± 3.79^a^	10.19 ± 3.81^a^	51.44 ± 89.71^b^	27.29 ± 15.46^ab^
DBIL(μmol/L)	3.15 ± 1.24^a^	3.95 ± 1.31^a^	39.36 ± 77.11^a^	13.13 ± 9.94^b^
IBIL(μmol/L)	5.02 ± 2.53^a^	8.66 ± 10.06^ab^	12.07 ± 13.19^b^	14.16 ± 9.59^b^
ALT(U/L)	25.19 ± 17.03^a^	36.95 ± 52.95^a^	335.87 ± 409.82^b^	46.56 ± 45.03^a^
AST(U/L)	21.4 ± 7.19^a^	26.38 ± 19.82^a^	193.58 ± 256^b^	70.69 ± 97.45^a^
AST/ALT	0.99 ± 0.32^a^	1.01 ± 0.51^a^	0.78 ± 0.44^a^	1.64 ± 1.05^b^
GGT(U/L)	25.79 ± 11.62^a^	26.48 ± 15.92^a^	138.42 ± 145.18^b^	86.54 ± 89.55^c^
ALP(U/L)	60.91 ± 14.75	67.52 ± 19.66	114.42 ± 80.4	94.26 ± 45.13
BUN (mmol/L)	5.46 ± 1.57^a^	4.89 ± 1.18^a^	3.82 ± 1.18^b^	5.02 ± 1.91^a^
SCR(μmol/L)	75.67 ± 13.12^ab^	79.7 ± 34.57^a^	71.21 ± 17.68^b^	71.46 ± 19.14^ab^
BUN/SCR	0.07 ± 0.02^ab^	3.98 ± 17.5^ab^	0.05 ± 0.02^b^	0.1 ± 0.13^a^
BUA(μmol/L)	339.87 ± 92.81^a^	339.65 ± 101.03^a^	279.89 ± 67.44^b^	301.94 ± 107.96^a^
TG (mmol/L)	1.38 ± 0.81^a^	1.12 ± 0.45^ab^	1.26 ± 0.55^ab^	0.98 ± 0.37^b^
CHOL (mmol/L)	5.11 ± 0.87^a^	4.72 ± 1.02^ab^	4.3 ± 1.56^b^	4.07 ± 1.56^b^
HDL (mmol/L)	1.43 ± 0.36^a^	1.35 ± 0.37^ab^	1.39 ± 0.52^a^	1.12 ± 0.29^b^
LDL (mmol/L)	3.24 ± 0.77^a^	3.2 ± 1.05^ab^	2.91 ± 2.01^ab^	2.65 ± 1.47^b^
FBG (mmol/L)	5.45 ± 0.49^a^	5.2 ± 1.18^ab^	5.08 ± 1.35^ab^	4.98 ± 1.52^b^
HbsAg (IU/ml)	0 ± 0^a^	7409.23 ± 17174.08^b^	4624.51 ± 6123.07^ab^	1585.75 ± 1326.34^a^
HBV DNA (10^7^ IU/mL)	0 ± 0^a^	1.77 ± 4.18^b^	11.0 ± 25.7^b^	0.42 ± 2.02^b^

*Letters indicate the ANOVA grouping among different HBV infection condition. Groups that do not share as common letter are significantly different. The four groups of patients according to clinical diagnosis included healthy, HBVI (chronic HBV carrier), CHB (chronic hepatitis B), and LC (liver cirrhosis) groups. WBC, white blood cell; RBC, red blood cell; Hb, hemoglobin; PLT, blood platelet; TP, total protein; ALB, albumin; GLO, globulin; ALB/GLO, albumin/globulin; TBIL, total bilirubin; DBIL, direct bilirubin; IBIL, indirect bilirubin; ALT, alanine aminotransferase; AST, aspartate aminotransferase; GGT, glutamyl transpeptidase; ALP, alkaline phosphatase; BUN, blood urea nitrogen; SCR, serum creatinine; BUN/SCR, blood urea nitrogen/serum creatinine; BUA, blood uric acid; TG, triglyceride; CHOL, cholesterol; HDL, high-density lipoprotein; LDL, low-density lipoprotein; and FBG, fasting blood glucose.*

The samples obtained from the same group were consistent compared with those across the groups (ANOSIM, *R* = 0.14, *p* = 0.001) (Supplementary Figure S1B). Moreover, the mantel test results (*r* = 0.15, *p* = 0.01) showed that the community difference might be due to the overall clinical distance difference, and not due to chance (Supplementary Figure S1C). The PERMANOVA results showed that disease stages (*R*^2^ = 0.08, *p* < 0.001) exerted a significant influence on the communities, while age (*R*^2^ = 0.03, *p* = 0.08), BMI (*R*^2^ = 0.03, *p* = 0.53) and gender (*R*^2^ = 0.01, *p* = 0.71) did not. Therefore, HBV infection progression itself was the primary driver of the alterations to the gut microbiota in this study (Supplementary Table S2). Ordination of all samples within the four HBV infection progression types indicated that their microbial communities were shaped primarily by factors associated with the infection state (ANOVA, *p* < 0.01) (Figure 1B). Site scores were significantly correlated with patient clinical variables, with changes observed in particular for RBC (envfit, *r*^2^ = 0.34, *p* < 0.001), Hb (envfit, *r*^2^ = 0.33, *p* < 0.001), ALB (envfit, *r*^2^ = 0.32, *p* < 0.001), PLT (envfit, *r*^2^ = 0.26, *p* < 0.001), and AST/ALT (envfit, *r*^2^ = 0.16, *p* < 0.01) (Supplementary Table S3). The compositional differences (OTUs) of individual fecal microbiota were graphed on NMDS plots, revealing that gut bacterial communities of the LC patients varied from the other groups (pairwise.adonis test, *p* < 0.05), and the differences were separated as HBV infection progressed by the first NMDS axis (Figure 1C). Further, the Bray–Curtis distance, based on genus composition, showed a similar trend (Figure 1D). These results indicate that the gut microbial communities changed in most of the HBV infected patients and most notably in the CHB or LC patients. Both the non-supervised and supervised ordination results therefore displayed the obvious gut microbiota dispersion associated with HBV infection progression.

### More Exclusive OTUs in HBV Infected Patients

**FIGURE 2:**
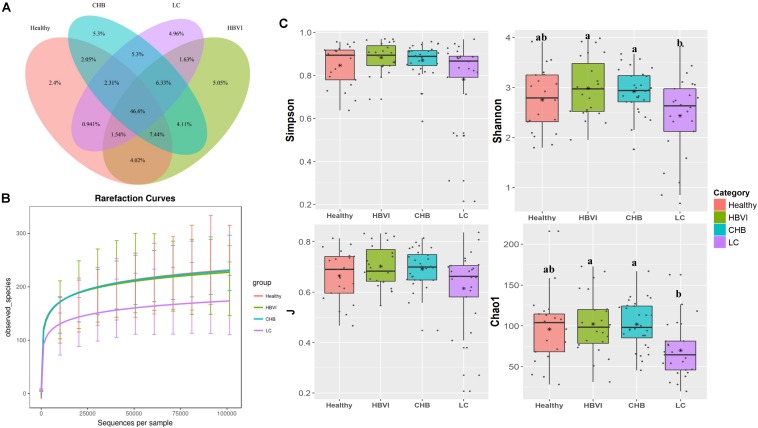
Comparisons of bacterial diversity among healthy and the HBV infection progression. **(A)** The Venn graph of percentage of shared and unique OTUs. **(B)** The observed species number comparisons. **(C)** The diversity indexes (Shannon, J, Simpson and Chao1) comparisons. Letters indicate the ANOVA groupings. The color scheme for Healthy, HBVI, CHB and LC in the graph are red, green, blue, and purple.

To investigate whether gut microbiota can be driven by HBV infection, we assessed the ratios of shared or unique OTUs in healthy controls and the HBVI, CHB, and LC patients. We found that the healthy group contained a lower proportion (2.4%) of exclusive OTUs, while the other HBV infected groups accounted for approximately 5% of the OTUs (Figure 2A), indicating that the HBV infected patients had more unique OTUs. Among the four groups, the healthy and HBVI groups had higher observed species than the CHB and LC groups (Figure 2B). Bacterial diversity (Shannon and Simpson), evenness (J) and estimated species (Chao1) were decreased in patients with LC compared with patients in earlier stages of the infection (Figure 2C) (ANOVA, *p* < 0.05). These results suggest that the lower bacterial diversity in the LC group might be due to unique taxa heterogeneity.

### Gut Microbiota Composition Changes as HBV Infection Progresses

**FIGURE 3:**
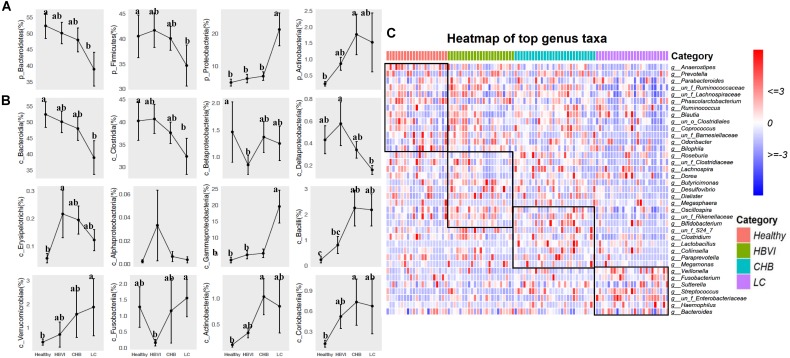
The bacterial composition of fecal samples from patients in different taxa levels. **(A)** Phyla distribution. **(B)** Class distribution. **(C)** Comparisons of top 40 genus taxa among four disease stages by heatmap. The color scheme for Healthy, HBVI, CHB and LC in the graph are red, green, blue, and purple.

To further demonstrate the microbiota shifts across HBV progression, we compared bacteria abundance between the groups. Bacteroidetes and Firmicutes decreased from healthy subjects, HBVI, CHB to LC patients, while Proteobacteria and Actinobacteria showed an upgrowing distribution (Figure 3A and Supplementary Figure S2A) (ANOVA, *p* < 0.05). The OTUs were classified into 32 classes, the top 12 of which occupied more than 99% of the abundance (Figure 3B). Within the Firmicutes class, the most abundant Clostridia decreased, while the Bacilli and Erysipelotrichi increased in the LC group (Figure 3B). These contrasting class patterns within the same phyla were also observed for Proteobacteria, within which the relative abundance of Gammaproteobacteria (average = 8.4%) increased to approximately 20% in the LC patients, even occupying >80% in two LC patients. The HBVI patients had lower Betaproteobacteria levels but were not different among the other three groups. The Deltaproteobacteria adversely correlated with the HBV infection progression while the least *Alphaproteobacteria* remained stable. *Actinobacteria, Verrucomicrobiae*, and *Coriobacteria* were more abundant in CHB and LC individuals, while *Fusobacteria* was the lowest in the HBVI patients (Figure 3B). Among the top 30 families, the LC patients had more *Pasteurellaceae, Enterobacteriaceae, Campylobacteraceae, Streptococcaceae*, and *Leptotrichiaceae*, and they had less *Odoribacteraceae, Rikenellaceae, Barnesiellaceae, Paraprevotellaceae, Clostridiaceae, Ruminococcaceae*, and *Moglibacteriaceae*. The CHB patients had more *Lactobacillaceae, Bifidobacteriaceae*, and *Coriobacteriaceae* (Supplementary Figure S2B). Among the top 40 genera, *Bacteroides* remained relatively stable in all four groups. *Haemophilus*, *Fusobacterium*, *Veillonella*, *Streptococcus*, and *Ruminococcus* were enriched in patients at a more serious stage of infection; however, *Dialister, Prevotella, Paraprevotella, Anaerostipes, Phascolarctobacterium, Butyricimonas*, and *Desulfovibrio* had higher distribution in the healthy individuals (Figure 3C, and Supplementary Figure S3).

### LEfSe Analysis to Screen out the HBV Infection-Related Bacterial Taxa

We further examined the differential OTUs in the HBV infected patients and the healthy controls using LEfSe. A total of 38 OTUs were assigned to the Bacteroidetes, Firmicutes, Fusobacteria, Proteobacteria and TM7 (phyla level), which were detected as significantly increased or decreased [log(LDA) > 2, *p* < 0.01]. Of these, 27 OTUs were enriched in the healthy group (Table 2). *Parabacteroides*, *Prevotella*, *Bacteroides*, and *Barnesiellaceae* from phyla *Bacteroidetes* were enriched in the healthy individuals, while distinguished genera within the S24-7 family had the opposite situation in both. *Ruminococcus* and *Ruminococcaceae* were significantly more abundant in the healthy individuals, while *Clostridium* and *Lactobacillus* decreased. In addition, *Sutterella* and *Desulfovibrio* were observed to be highly abundant in the HBV infected patients. Interestingly, *Fusobacterium* within Fusobacteria was significantly more (almost 10 times) abundant in the HBV infected patients than in the healthy individuals.

**TABLE 2:** Differential OTUs selected by linear discriminant analysis effect size (LEfSe).

OTUID	Phyla	Genus	Enriched group	LDA	*p*-value	HBV_mean	Healthy_mean
OTU637	Bacteroidetes	*Bacteroides*	Healthy	2.19	0.03	2.85E-04	1.12E-03
OTU26	Bacteroidetes	*Parabacteroides*	Healthy	3.06	0.04	5.22E-2	5.33E-02
OTU181	Bacteroidetes	*Prevotella*	Healthy	2.57	0.01	6.60E-03	1.31E-02
OTU200	Bacteroidetes	*Prevotella*	Healthy	2.79	0.00	1.45E-03	1.88E-03
OTU733	Bacteroidetes	*Prevotella*	Healthy	2.05	0.00	6.60E-05	2.46E-03
OTU109	Bacteroidetes	un_f_Barnesiellaceae	Healthy	3.29	0.04	2.61E-02	4.52E-02
OTU301	Bacteroidetes	un_f_Barnesiellaceae	Healthy	2.78	0.02	5.24E-03	1.20E-02
OTU1038	Bacteroidetes	un_f_S24-7	Healthy	2.17	0.00	6.60E-05	5.02E-04
OTU255	Bacteroidetes	un_f_S24-7	Healthy	2.80	0.05	1.19E-03	5.65E-03
OTU286	Bacteroidetes	un_f_S24-7	HBV	2.75	0.01	2.40E-03	2.51E-04
OTU385	Bacteroidetes	un_f_S24-7	Healthy	2.41	0.01	6.60E-05	5.01E-03
OTU407	Bacteroidetes	un_f_S24-7	HBV	2.33	0.03	1.42E-03	1.14E-03
OTU764	Bacteroidetes	un_f_S24-7	Healthy	2.02	0.03	1.32E-04	1.12E-03
OTU83	Firmicutes	*Clostridium*	HBV	2.77	0.05	1.14E-02	8.86E-03
OTU252	Firmicutes	*Lactobacillus*	HBV	2.35	0.05	3.18E-03	5.02E-04
OTU254	Firmicutes	*Lactobacillus*	HBV	2.45	0.04	3.51E-03	0
OTU282	Firmicutes	*Megasphaera*	Healthy	2.52	0.05	0	5.62E-03
OTU380	Firmicutes	*Megasphaera*	Healthy	2.58	0.01	9.59E-04	5.83E-03
OTU337	Firmicutes	*Phascolarctobacterium*	HBV	2.56	0.01	2.54E-03	1.05E-03
OTU147	Firmicutes	*Ruminococcus*	Healthy	2.54	0.02	8.00E-03	1.42E-02
OTU242	Firmicutes	*Ruminococcus*	HBV	2.52	0.03	1.56E-03	7.10E-04
OTU244	Firmicutes	*Ruminococcus*	Healthy	2.25	0.01	4.02E-03	5.39E-03
OTU638	Firmicutes	*Ruminococcus*	Healthy	2.03	0.03	2.53E-04	7.89E-04
OTU523	Firmicutes	un_f_Christensenellaceae	Healthy	2.11	0.01	3.57E-04	1.68E-03
OTU696	Firmicutes	un_f_Mogibacteriaceae	HBV	2.00	0.01	5.05E-04	0
OTU1204	Firmicutes	un_f_Ruminococcaceae	Healthy	2.03	0.03	4.23E-04	8.57E-04
OTU567	Firmicutes	un_f_Ruminococcaceae	Healthy	2.02	0.01	4.44E-04	1.92E-03
OTU579	Firmicutes	un_f_Ruminococcaceae	Healthy	2.02	0.03	5.38E-04	1.21E-03
OTU624	Firmicutes	un_f_Ruminococcaceae	Healthy	2.25	0.01	1.32E-04	3.55E-04
OTU796	Firmicutes	un_f_Ruminococcaceae	Healthy	2.08	0.01	1.75E-04	2.51E-04
OTU779	Firmicutes	un_f_Veillonellaceae	Healthy	2.08	0.01	1.14E-04	2.51E-04
OTU801	Firmicutes	un_o_Clostridiales	Healthy	2.09	0.01	0	7.10E-04
OTU847	Firmicutes	un_o_Clostridiales	Healthy	2.19	0.00	1.32E-04	2.51E-04
OTU113	Fusobacteria	*Fusobacterium*	HBV	3.05	0.03	1.67E-02	1.94E-03
OTU104	Proteobacteria	*Desulfovibrio*	HBV	3.18	0.01	1.29E-02	1.05E-02
OTU263	Proteobacteria	*Sutterella*	Healthy	2.74	0.00	1.07E-03	6.51E-03
OTU268	Proteobacteria	*Sutterella*	HBV	2.29	0.01	1.64E-03	1.29E-03
OTU560	TM7	un_c_TM7-3	Healthy	2.05	0.00	3.57E-04	8.57E-04

**p*-values were adjusted with FDR method.*

### Indicators Analysis Associated the HBV Infection Progression

**FIGURE 4:**
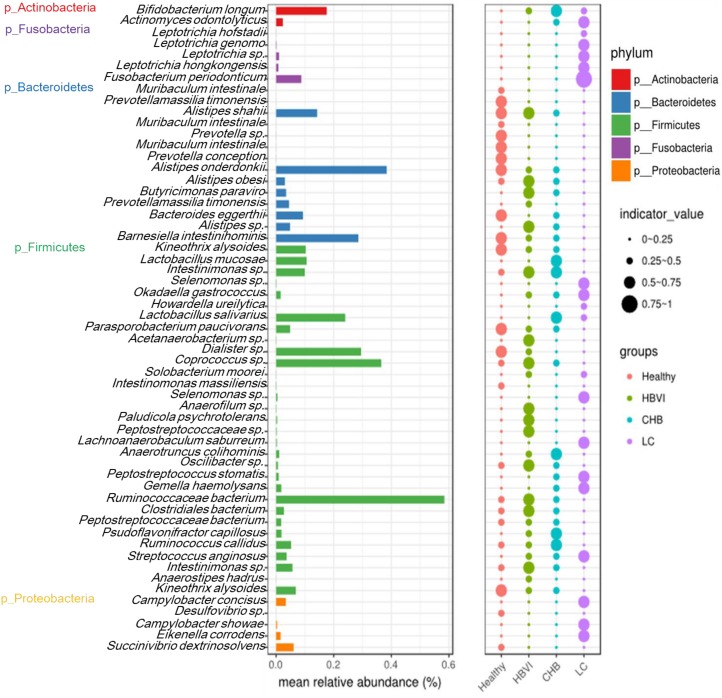
Indicator species significantly (*q* < 0.1) associated with the disease progression. The bar indicates the relative abundance of each indicator species, while the size of each circle represents the indicator value (association strength) of a specific species with the different group: 0–0.25, not characteristic; 0.25–0.5, weakly characteristic; 0.5–0.75, characteristic; and 0.75–1.0, strongly characteristic. The color scheme for Healthy, HBVI, CHB and LC in the graph are red, green, blue, and purple.

Indicator analysis identified 57 OTUs that were significantly (qvalue, *q* < 0.1) associated with infection progression, of which 32 OTUs belonged to Firmicutes. The most abundant OTU was classified as OTU21 (Ruminococcaceae bacterium), which occupied approximately 0.6%, while OTU50 (*A. onderdonkii*), OTU48 (*Coprococcus* sp.) and OTU51 (*D. succinatiphilus*) followed in abundance. The OTUs with the highest indicator value (0.75∼1) were classified as OTU113 (*Fusobacterium periodonticum*), which occurred in the LC group (Figure 4).

**FIGURE 5:**
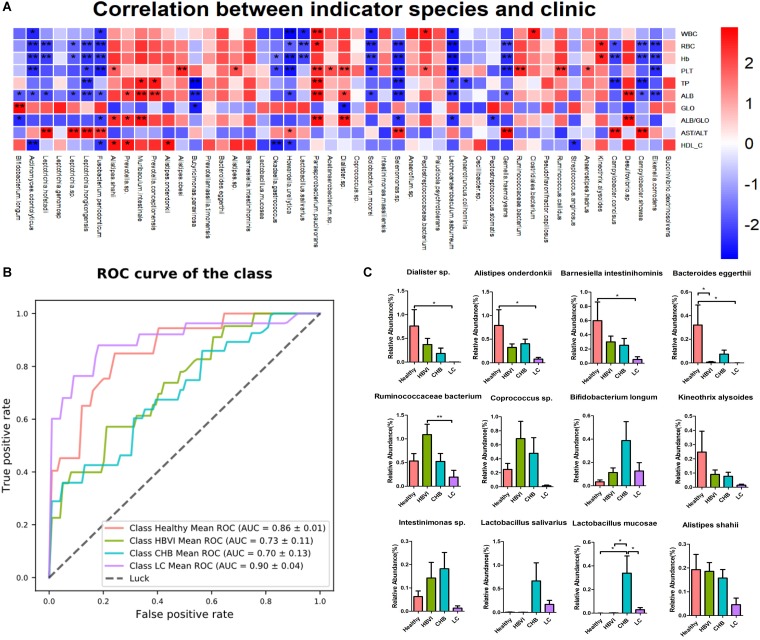
The indicator species’ distribution and correlation with clinics along with the disease progression. **(A)** The correlation graph between clinics and indicator species, * denotes significance *p* < 0.05 and ** denotes *p* < 0.01. **(B)** The ROC curve based on the indicator OTU to predict its potential to distinguish healthy and non-healthy, HBVI and non-HBVI, CHB and non-CHB, LC and non-LC. **(C)** The distribution of those indicator species with higher abundance (>0.1%) that significantly correlated with clinics. * Denotes significance *p* < 0.05 and ** denotes *p* < 0.01. The color scheme for Healthy, HBVI, CHB and LC in the graph are red, green, blue, and purple.

The correlation between the 57 indicator OTUs and the 10 significant clinical parameters were further evaluated to screen out those specific OTUs that might tightly relate with clinic applications. The results showed that OTU182 (*Eikenella corrodens*), OTU226 (*Gemella haemolysans*), OTU199 (*Leptotrichia hongkongensis*), OTU226 (*Leptotrichia hofstadii*), OTU308 (*Leptotrichia* sp.), OTU113 (*F. periodonticum*), OTU776 (*Selenomonas* sp.), OTU341 (*Lachnoanaerobaculum saburreum*), OTU646 (*Howardella ureilytica*), OTU315 (*Actinomyces odontolyticus*), and OTU678 (*Okadaella gastrococcus*), OTU467 (*Solobacterium moorei*) were adversely associated with WBC, RBC, PLT, Hb, TP, ALB, ALB/GLO, and HDL changes, but were positively associated with GLO and AST/ALT levels. The opposite correlation was observed for OTU119 (*Bacteroides eggerthi*), OTU51 (*D. succinatiphilus*), OTU109 (*Barnesiella intestinihominis*), OTU50 (*A. onderdonkii*), OTU21 (*Ruminococcaceae bacterium*), OTU108 (*Kineothrix alysoides*), OTU841 (*Muribaculum intestinale*), OTU81 (*Alistipes shahii*), OTU627 (*Prevotella conceptionensis*), and OTU570 (*Parasporobacterium paucivorans*) (Figure 5A).

An AUC value of 90% (95% CI: 86–94%) between the LC and non-LC groups was obtained with a combination of bacterial indicator markers, followed by its potential in distinguishing the healthy individuals from patients (Figure 5B). In addition, based on the correlation significance and relative abundance (>0.1%), 12 OTUs were screened out to investigate their relative abundance distribution along the HBV infection process. Their taxonomic information displayed increasing or decreasing trends in accordance with disease progression. For example, the relative abundance of OTU51 (*D. succinatiphilus*), OTU50 (*A. onderdonkii*), OTU109 (*B. intestinihominis*), OTU119 (*B. eggerthii*), OTU108 (*K. alysoides*), and OTU81 (*A. shahii*) gradually decreased as the disease progressed to LC (Figure 5C). In addition, the relative abundance of OTU50 (*A. onderdonkii*) and OTU51 (*D. succinatiphilus*) displayed similar trends than that of the 16S rDNA analysis by qPCR in another set of subjects (which also covered healthy to LC patients) (Supplementary Figure S4), which indicated that they might be beneficial in inhibiting the progression of liver disease caused by HBV infection.

### More Complexed Network Features in LC Patients

**FIGURE 6:**
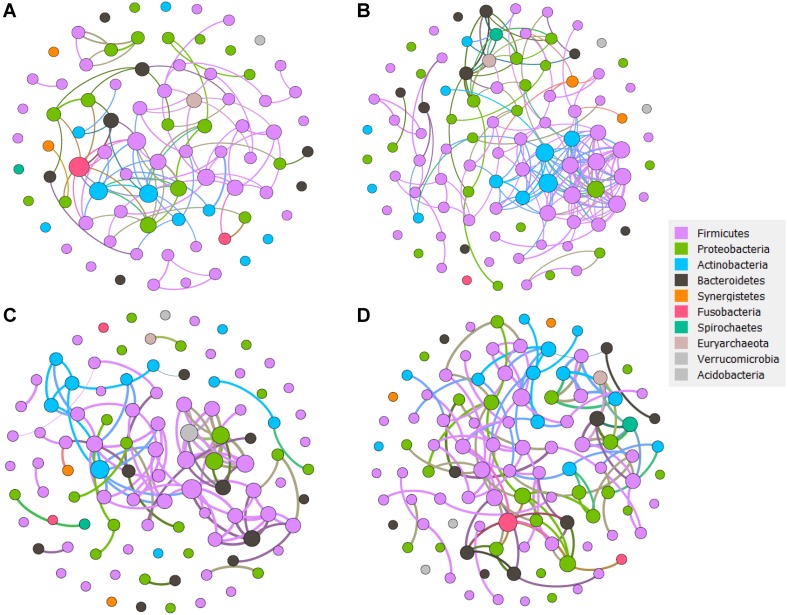
The network analysis revealing the co-occurrence patterns between bacterial taxa for each sample group. **(A)** Healthy group network. **(B)** HBVI group network. **(C)** CHB group network. **(D)** LC group network. The nodes were colored according to the phyla assigned by the taxa. A connection represents a strong (Spearman’s correlation coefficient *R*^2^ > 0.6) and significant (*p* < 0.05) correlation. Edges weighted according to the correlation coefficient and node size weighted according to the relative abundance of microbial taxa.

Correlation networks of each patient group that are directly related to disease progression, are well suited to detect the general patterns in highly populated taxonomic groups (Figure 6, and Supplementary Tables S4, S5). We hypothesized that non-random cooccurrence patterns between taxa are present if the coexisting microbial taxa possessed a strong and significantly positive correlation (cor.test, *R*^2^ > 0.6, *p* < 0.05). For the healthy group, whose modularity, nodes, and edges were 0.69, 81 and 96 separately, *Leptotrichia* from Fusobacteria, *Enterococcus* and *Ruminococcus* from Firmicutes, *Actinobacillus* from Proteobacteria were more connected. In the HBVI group, the nodes that clustered together mainly included *Anaerococcus*, *Staphylococcus*, *Geobacillus*, *Succinivibrio*, and *Peptoniphilus*. In the CHB group, *Peptoniphilus*, *Rhodoplanes*, *Burkholderia*, *Staphylococcus*, *Corynebacterium*, *Peptococcus*, *Abiotrophia*, and *Lactobacillus* were the taxa that centered with the others. In the LC group, *Leptotrichi*a, *Catenibacterium*, *Actinomyces*, *Acidaminococcus*, *Burkholderia*, *Peptoniphilus*, *Fusobacteria*, and *Bulleidia* dominated the network and were more connected with the other bacterial taxa. As the disease progressed from the healthy subjects to the LC group, the correlation networks became more complex.

## Discussion

In this study, we investigated the association between HBV infection progression and gut microbiota changes in a larger number of HBV infected patients, which covered HBVI, CHB, and LC patients. The results suggest that gut bacterial diversity significantly decreased in the LC subjects compared with the other groups. Furthermore, the gut microbiota communities significantly correlated with clinical parameters including WBC count, RBC count, PLT count, Hb, TP, ALB, ALB/GLO, and HDL levels, which may be closely related to liver fibrosis. In addition, indicator analyses identified featured bacterial taxa characteristic during HBV infection progression which could serve as a model to predict disease progression. The qPCR verification results verified the distribution of *D. succinatiphilus* and *A. onderdonkii*. Moreover, to identify bacterial assemblages that potentially interact or share niches within HBV infection progression, we constructed cooccurrence networks and found that gut microbial network connectivity and complexity increased during disease progression.

### Gut Dysbiosis Closely Correlated With Clinical Features

The significant decrease of α diversity with disease progression, which was especially significant in LC patients, was revealed in previous studies using 16S rRNA (Wu et al., 2012; Wiest et al., 2014; Mullish et al., 2018) and metagenome sequencing (Qin et al., 2014). Fecal samples were collected and matched according to age (Chou et al., 2015), which were reported to influence HBV clearance and gut microbiota population. Regarding the clinical features, only peripheral WBC count, RBC count, PLT count, and Hb changes were greatly affected by the hypersplenism caused by liver fibrosis. TP, ALB, and ALB/GLO levels, which reflect liver function, and HDL protein synthesis, which is closely related to cholesterol transport, had higher positive correlation with the db-RDA1 axis (Figure 1B). In contrast, GLO and AST/ALT which reflect severe liver damage, were negatively correlated with the db-RDA1 axis. However, there was no significant correlation between TBIL, DBIL, IBIL, ALT, AST, HbsAg, and HBV DNA and the overall structure of gut microbial communities, which indicated that although these indices were changed, temporary inflammation of liver or HBV replication is not sufficient to cause dramatic changes in the overall gut microbiota. OTUs were classified into four major phyla, including Bacteroidetes, Firmicutes, Proteobacteria and Actinobacteria (each more than 1% of the total sequences). In addition, we revealed that Bacteroidetes, *Parabacteroides* decreased while Proteobacteria increased in the patients. It has been confirmed that these bacteria are restored to levels similar to levels found in healthy individuals after splenectomy, which was accompanied with and upregulation in WBC and PLT counts in liver cirrhosis patients (Liu et al., 2018). Bacteroidetes and Firmicutes decreased, while Proteobacteria increased during chronic HBV infection. A similar trend has been found during NAFLD, while the change in Actinobacteria showed the opposite trend, and increased during chronic HBV infection (Del Chierico et al., 2017).

### Lower Diversity and Higher Network Complexity in LC

Network analysis is often used to reveal non-random covariation patterns that may reflect community organization and can provide a tool to reveal microbe interactions and the core bacterial taxa that are difficult to assess in microbial communities in different application conditions (Kelder et al., 2014; Sung et al., 2017; Bauer and Thiele, 2018). For example, Shi et al. (2016) used network complexity, which was a previously undescribed property of the rhizosphere microbiome, to define characteristics of the plant rhizosphere microbial activity and found that community organization is not captured by univariate diversity. However, there were no previously identified network analysis results associated with HBV progression. In this study, more nodes within Firmicutes were highly connected, and they were accompanied by a higher bacterial diversity than that of the other HBV infection groups. As the HBV infection progressed, the network complexity was accompanied with decreased bacterial diversity and fewer nodes within Firmicutes; however, more nodes within Actinobacteria were observed. We considered that even though the bacteria in the healthy groups were numerically more abundant than those in the LC patients, the bacterial communities in the patients with severe HBV infection were less rich because particular disease-related taxa became dominant in the HBV infection related disease patients. As *D. succinatiphilus A. onderdonkii, B. intestinihominis, K. alysoides, A. shaii*, and *B. eggerthii* decreased when evaluating the spectrum from healthy individuals to LC patients, we postulated that bile acid or other metabolites produced from microbial communities may promote the development of niches populated by dominant taxa, which would concurrently yield decreased diversity and greater interactions and would overall result in more complex cooccurrence patterns along with the HBV infection progression. The inverse relationship between diversity and network connectivity highlight the importance of studying the relationships among organisms, as they are a crucial dimension of community organization not captured by univariate diversity metrics.

### The Role of Indicator OTUs in the Progression of HBV Infection

Although severe gut dysbiosis, which is associated with hypersplenism and great liver dysfunction, occur in the LC stage of HBV related liver disease, downregulation of *D. succinatiphilus*, *A. onderdonkii*, *B. intestinihominis*, *A. shahii*, *K. alysoides*, and *B. eggerthii* occurred even in the earliest phase of HBV infection. Combining the abundance of these bacteria with serum HBV DNA, serum ALT changes in patients may help predict the risk of disease progression and determine the timing of antiviral therapy or evaluate curative effects. Early diagnosis and treatment toward compensated cirrhosis may help delay cirrhosis from the compensated to the decompensated state. Ren et al. (2018) reported that the gut microbiome could serve as a tool to target noninvasive biomarkers for early hepatocellular carcinoma (Bernardi and Caraceni, 2018). Qin previously established an accurate patient discrimination index based on 15 microbial biomarkers and found that microbiota-targeted biomarkers could serve as a powerful tool for an LC diagnosis (Qin et al., 2014). In this study, a combination of bacterial indicator markers distinguished the LC and non-LC groups, which provides a new method for the early diagnosis of LC to guide clinical treatment.

In this study, we found that OTUs positively associated with worsening clinical parameters, which reflected hepatic decompensation. Several OTUs were enriched in the late stage of LC and were all of oral origin, including *E. corrodens, G. haemolysans, Leptotrichia, F. periodonticum, Selenomonas* sp., *A. odontolyticus*, and *S. moorei*, which have been reported to cause opportunistic infections, such as a liver abscess or endocarditis, or have been associated with colorectal cancer (Malik et al., 2010; Woo et al., 2010; Chao et al., 2011; Warren et al., 2013; Revest et al., 2016; Eriksson and Lif, 2017; Yu et al., 2017; de Carvalho Baptista et al., 2018). Intestinal colonization of bacteria of oral origin has been associated with many diseases. Atarashi et al. (2017) reported that oral bacteria *Klebsiella* sp. induced T helper 1 cells and triggered gut inflammation when it colonized the gut. Furthermore, antibiotic resistance of oral bacteria makes it difficult to cure opportunistic infections caused by oral bacteria invading through the fragile intestinal mucosal barrier during LC. The oral bacteria enriched in the LC group may be potential pathogens of opportunistic infection or a stimulator of inflammation in cirrhosis, and may cause severe damage to the organism. The diminished bactericidal effects of gastric acid, overgrowth of intestinal bacteria and gut dysbiosis in patients may contribute to the colonization of oral bacteria in the gut (Qin et al., 2014).

In addition, we found that the OTUs gradually decreased when evaluating the spectrum from healthy individuals to LC patients, and these OTUs have been reported to contribute to immunity function and the maintenance of gut mucosal barrier function, which play an important role in reducing translocation of bacterial products. *Alistipes* and *Prevotella* decreased in NAFLD compared with healthy subjects, and this change was accompanied by a downregulation of CD4+ and CD8+ T cells, an upregulation of TNF-α, IL-6, IFN-γ, irregularly arranged microvilli, and widened tight junctions in gut mucosa (Jiang et al., 2015). *B. intestinihominis* facilitated cyclophosphamide-induced therapeutic immunomodulatory effects *via* the promotion of the IFN-γ producing γδT cell infiltration in cancer lesions in advanced lung and ovarian cancer patients (Daillère et al., 2016). Butyrate-producing bacteria *K. alysoides* could facilitate maintenance of the intestinal barrier by providing an energy source for enterocytes (Haas and Blanchard, 2017). Downregulation of these enriched bacteria in healthy individuals may promote the colonization of pathogens in the gut and further promote systemic inflammation and worsen hepatic dysfunction, which could cause greater gut dysbiosis *via* intestinal congestion or bile excretion disorder, forming a vicious circle. Restoration of gut dysbiosis may break this circle.

### FMT as a Tool to Intervene Gut Dysbiosis Related With HBV Infection

Treatment of liver diseases that target gut microbiota has been confirmed to effectively improve clinical indicators. Bajaj et al. improved cognition and dysbiosis in cirrhosis with recurrent hepatic encephalopathy (HE) by transplanting fecal microbiota from a rational stool donor to patients that may benefit from a reduction of ammoniacal bacteria (Mullish et al., 2017). Bajaj et al. (2018b) revealed that FMT restored antibiotic-associated disruption in microbial diversity and function when the donor sample was enriched in Lachnospiraceae and Ruminococcaceae, which were highly abundant in the healthy subjects and less abundant in LC patients in our study. Ren et al. (2017) induced HBeAg clearance in patients with positive HBeAg after long-term antiviral therapy, which was accompanied by an increasing relative abundance of *Prevotella* and *Lachnospiraceae*. In this study, we observed gut microbiota alterations in earlier stages of chronic HBV infection, such as HBVI and CHB, while the most serious alterations were observed in the LC patients. Interestingly, the relative abundance of *A. onderdonkii* and *D. succinatiphilus* were highest in the healthy individuals and low in the HBV infected patients, which were both revealed by our 16S rRNA sequencing and qPCR results. *A. onderdonkii*, which can be isolated from human intestines, is strictly anaerobic and produces succinic acid from the principal metabolic end-product of glucose fermentation (Yuli et al., 2006). Results have shown that *Alistipes* were decreased in CHB patients compared with healthy controls (Wang et al., 2017). Therefore, *A. onderdonkii* might help to activate the immune response to cure HBV patients by releasing succinic acid, which has been shown to have beneficial effects (Ladrière et al., 1998; Xu and Guo, 2010; Iplik et al., 2018). *D. succinatiphilus* has been isolated from human feces and grows in anaerobic conditions, and is able to decarboxylate succinate to propionate (Morotomi et al., 2008), which can bind to the GPR-43 that is expressed on lymphocytes in order to maintain an appropriate immune defense (Cani, 2018).

This study covered the HBV infection process, from HBVI to LC, and aimed to reveal the unique microbiota profile during each disease stage, which might help the future remission of liver disease through intervening gut microbiota treatments. However, our research has several limitations. First, the results need further multicenter verification, consisting of several hospitals with the same patient inclusion standards. Second, it is essential to understand the details of how the differences in HBV genotypes affect the gut microbiota due to their pathogenicity. Third, the reason gut dysbiosis exists in chronic HBV infection is not well known and should be explored in further studies. Fourth, the reason *D. succinatiphilus* and *A. onderdonkii* decrease while *F. periodonticum* increases in HBV infected patients is not completely understood and should be examined and discussed in further studies.

## Conclusion

In summary, we investigated gut microbiota alterations between healthy HBVI, CHB, and LC subjects. *Prevotella* and *Phascolarctobacterium* were the least abundant in the LC group; however, they were the most abundant in the healthy group. *Fusobacteria, Klebsiella, Veillonella* and *Haemophilus* increased as the disease progressed. In addition, the clinical features and gut microbiota communities showed significant linear relationships, especially with changes in RBC, Hb, PLT, AST/ALT, and ALB levels. The significantly higher abundance of *D. succinatiphilus* and *A. onderdonkii* in the healthy cohort, while becoming less in the more serious disease stages, imply their potential benefit and requires further in-depth investigations. The complexed concurrence network patterns associated with the infection progression, demonstrated that ecology networks formed by specific bacterial taxa with detrimental features were the key-stone taxa in HBV progression, and their reversion might be attainable *via* beneficial taxa, such as *D. succinatiphilus* and *A. onderdonkii*.

## Data Availability Statement

The datasets generated for this study can be found in NCBI SRA submission (SUB6109752), BioProject (PRJNA558158).

## Ethics Statement

This study was performed in accordance with the approved guidelines, the Ethics Committee of Xiamen University with written informed consent from all subjects (Permit Number ID: XMU-IRB-2018001) and also registered in ClinicalTrials.gov (ID:NCT03587467). All participants provided informed consent, and the study protocol conformed to the ethical guidelines of the Declaration of Helsinki.

## Author Contributions

ZC, YX, and FZ conceived and designed the experiments. FZ, JW, and QC collected the fecal samples. YX analyzed the clinical data. ZC and LY extracted the DNA. ZC, BZ, and SX analyzed the sequencing data. ZC, YX, and XZ wrote the manuscript. ZC, JL, RS, and XZ corrected the manuscript. XZ, HX, and JR decided to publish. All authors read and approved the final version of the manuscript.

## Conflict of Interest

The authors declare that the research was conducted in the absence of any commercial or financial relationships that could be construed as a potential conflict of interest.

## Footnotes

1 https://www.qiagen.com/cn/shop/sample-technologies/dna/genomic-dna/qiaamp-powerfecal-dna-kit

2 http://www.novogene.com/

3 https://www.ncbi.nlm.nih.gov/tools/primer-blast/

4 http://scikitlearn.org/

5 https://github.com/zhangranluxi/HBV
