# Supplementary material for: Featured Gut Microbiomes Associated With the Progression of Chronic Hepatitis B Disease
Source: Front Microbiol. 2020 Mar 20;11:383. doi: 10.3389/fmicb.2020.00383 (PMC7098974; doi:10.3389/fmicb.2020.00383)
Supplement: Supplementary file 1 [file Data_Sheet_1.docx]

Supplementary Material

## Supplementary Figures


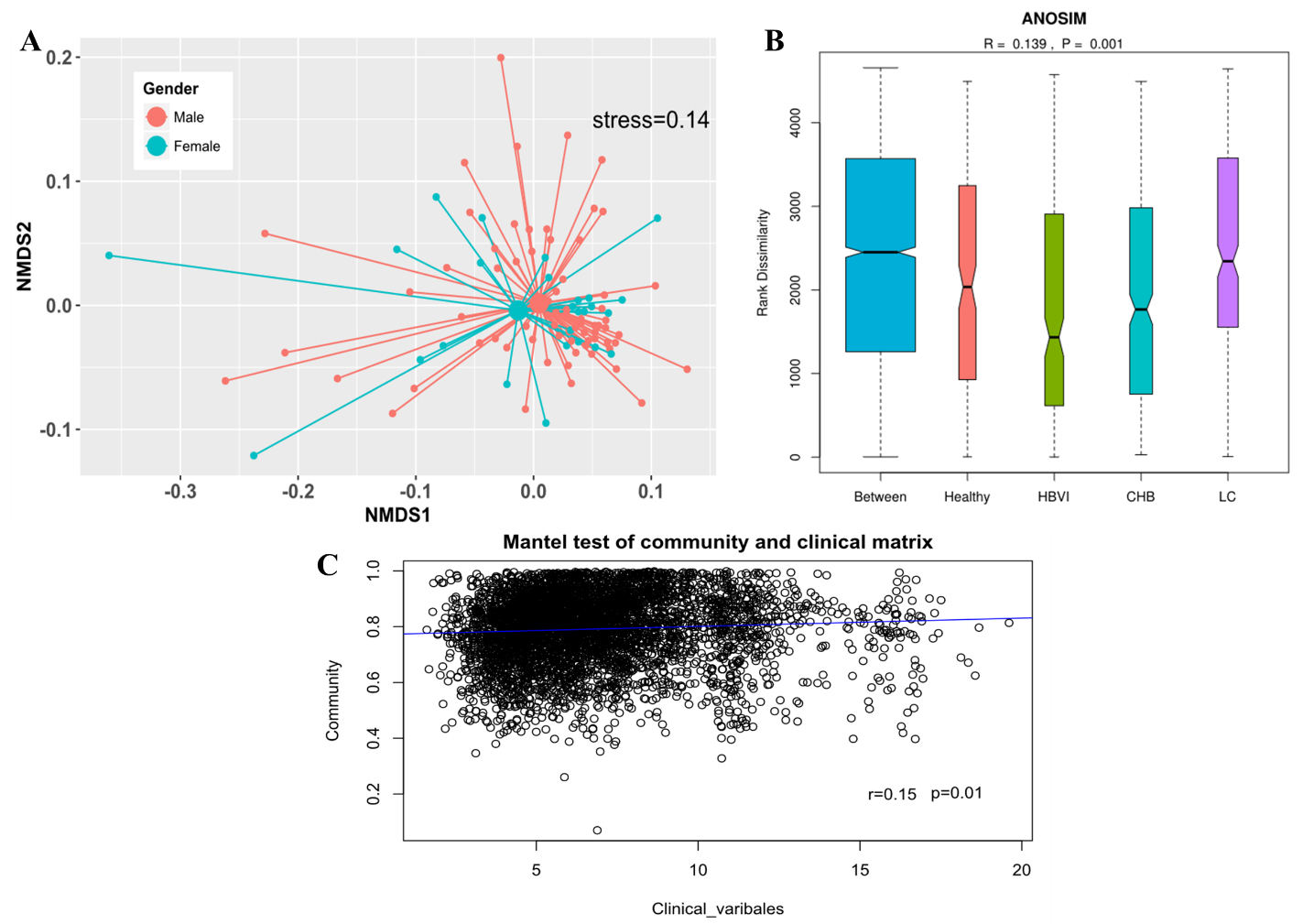


**Supplementary Figure 1.** (A) Non-metric multidimensional scaling (NMDS) based on the Bray-Curtis distance showing the overall distribution grouped by gender. (B) The ANOSIM analysis of bacterial community based on the OTU within and between groups. (C) The mantel test of community distance and clinical distance matrix.


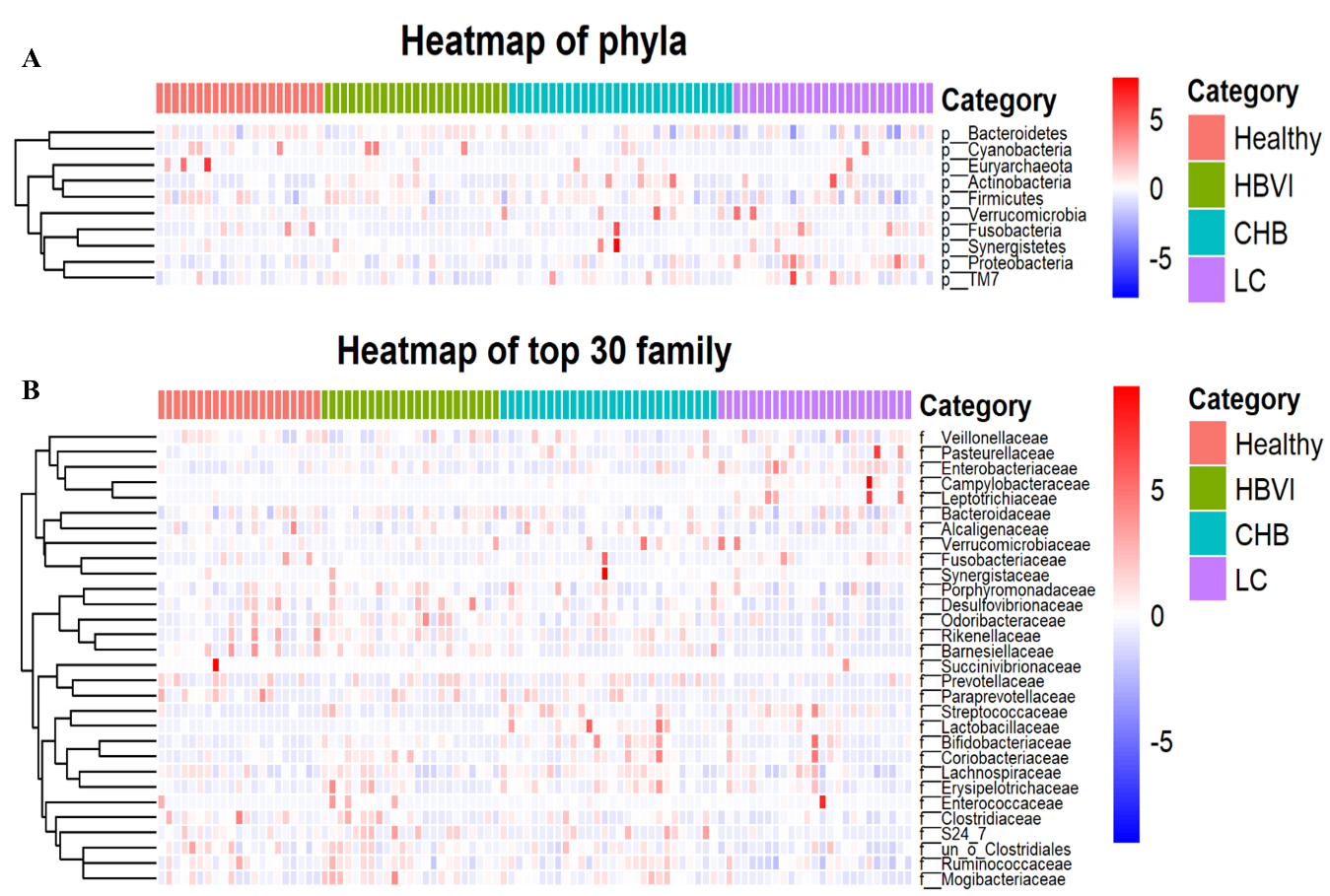


**Supplementary Figure 2.** Heatmap of gut microbiota taxa in phyla and family level. (A) Heatmap of 10 detected bacterial phyla distribution within four groups. (B) Heatmap of the top 30 family with higher abundance.


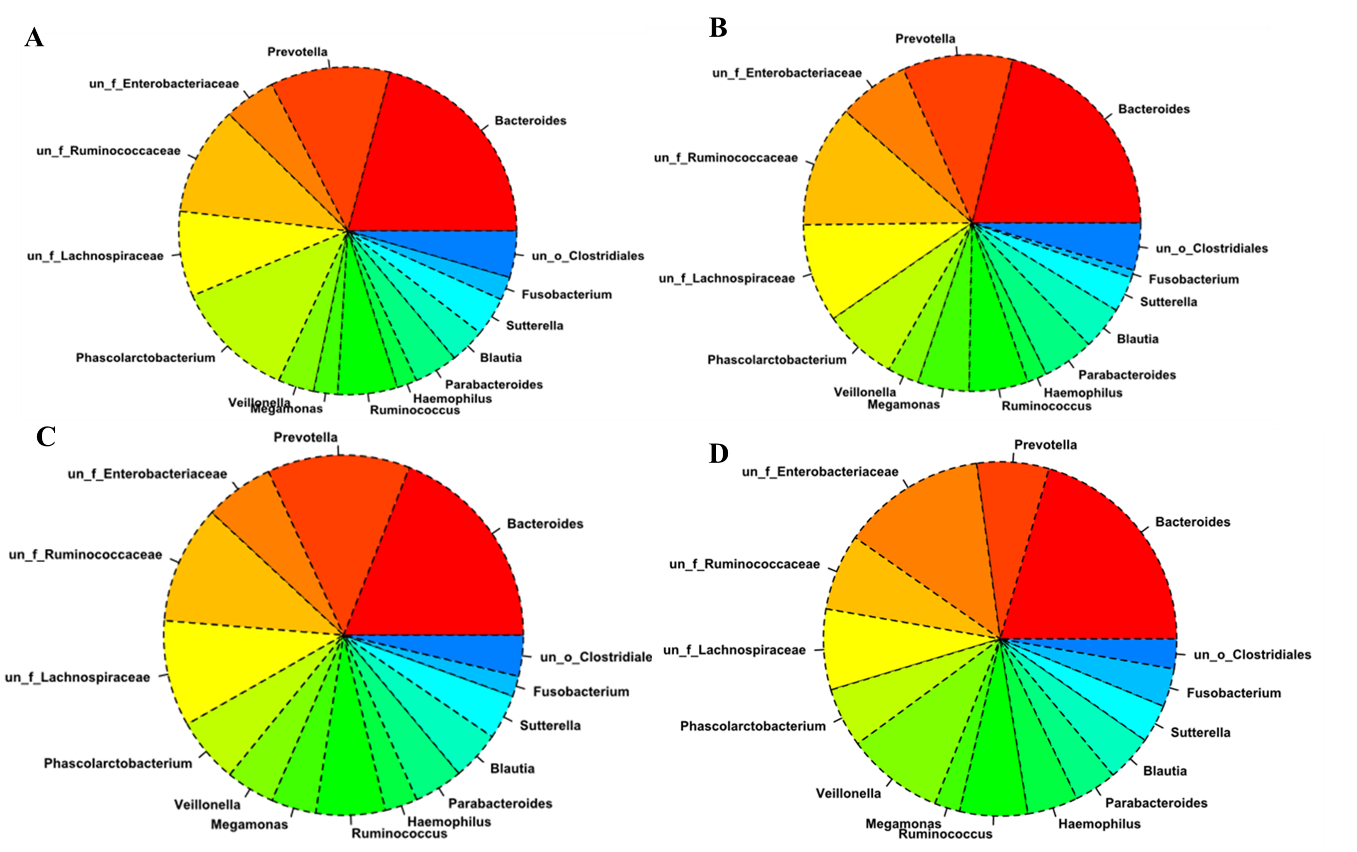


**Supplementary Figure 3.** Genus composition of fecal samples from healthy individuals and hepatitis B virus infection patients. Fecal samples from (A) healthy individuals (n=21) and HBV infection patients diagnosed as (B) HBV carrier (n=23), (C) chronic hepatitis B (n=28) and (D) liver cirrhosis (n=25). The circle graphs show the bacterial balance of gut microbiome in each group.


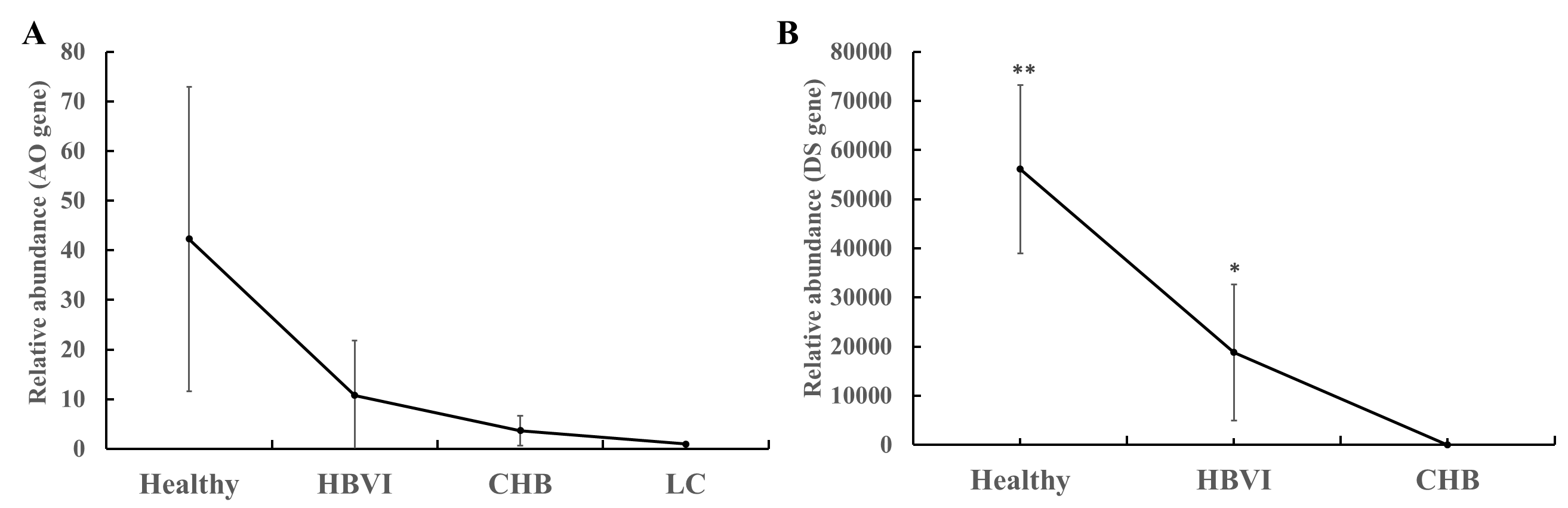


**Supplementary Figure 4.** Relative abundance of *Alistipes onderdonkii*.(A) and *Dialister succinatiphilus* (B) detected by qPCR.
